# DSSA-TCN: Exploiting adaptive sparse attention and diffusion graph convolutions in temporal convolutional networks for traffic flow forecasting

**DOI:** 10.1371/journal.pone.0336787

**Published:** 2025-11-13

**Authors:** Zhouyuan Zhang, Xin Wang, Xu Tan, Jiatian Pi

**Affiliations:** 1 School of Computer and Information Science, Chongqing Normal University, Chongqing, China; 2 Chongqing Changan Automobile Company Limited, Chongqing, China; 3 China Telecom Zhi’an Technology Co., Ltd., Chongqing, China; 4 National Center for Applied Mathematics in Chongqing, Chongqing Normal University, Chongqing, China; National University of Defense Technology, CHINA

## Abstract

Accurate traffic flow forecasting is essential for intelligent transportation systems, yet the nonlinear and dynamically evolving spatio-temporal dependencies in urban road networks make reliable prediction challenging. Existing graph-based and attention-based approaches have improved performance but often decouple spatial and temporal learning, which leads to redundant computation and weak directional interpretability. To address these limitations, we propose DSSA-TCN, a unified framework that establishes an alternating spatio-temporal coupling mechanism, where each temporal convolutional block is tightly integrated with an adaptive spatial module that combines sparse attention with diffusion-based graph convolution. Within this mechanism, adaptive sparse attention dynamically selects the most informative neighbors to reduce spatial complexity, and bidirectional diffusion convolution enforces physically consistent directional and multi-hop propagation over the road topology. Temporal patterns are modeled with gated dilated convolutions to preserve parallelism and stability. Comprehensive experiments on six real-world datasets demonstrate that DSSA-TCN achieves superior forecasting accuracy and computational efficiency while providing interpretable spatial reasoning. These results indicate that layer-wise coupling of adaptive sparsity and diffusion within a causal temporal backbone offers a scalable and physically grounded paradigm for spatio-temporal traffic prediction.

## Introduction

Traffic flow forecasting plays a pivotal role in intelligent transportation systems (ITS), enabling key applications such as adaptive traffic signal control, dynamic route planning, and urban congestion mitigation [1]. With the rapid pace of urbanization and the growing complexity of transportation networks, there is an increasing demand for accurate and efficient predictive models to support data-driven decision-making in modern traffic management [2].

Traffic data inherently exhibit complex patterns across both temporal and spatial dimensions. From a temporal perspective, traffic flow is shaped by human mobility behaviors and infrastructure schedules, leading to strong short-term autocorrelation and recurring periodic trends, such as daily rush hours or weekly commuting pattern [3]. This phenomenon is further supported by large-scale empirical evidence. Chang *et al*. [4] examined metro travel data from multiple Chinese cities and revealed hierarchical and periodic regularities through travel-motif and entropy-based analyses, demonstrating that traffic dynamics are shaped by collective behavioral patterns and spatial structures. However, traffic dynamics can also undergo abrupt changes due to unexpected incidents, including traffic accidents, road closures, or adverse weather conditions, making temporal dependencies highly nonlinear and context-sensitive [5]. From a spatial perspective, traffic sensors are deployed over non-Euclidean road networks, where interactions among road segments are inherently irregular, directional, and governed by physical road topology, lane configurations, and traffic regulations [6]. These temporal and spatial dependencies are tightly intertwined, posing a fundamental challenge for developing unified models capable of capturing both local and global dynamics in complex transportation systems.

Traditional traffic forecasting methods, such as historical averages and Auto-Regressive Integrated Moving Average (ARIMA) [7], offer computational efficiency and interpretability. However, they are fundamentally incapable of modeling the nonlinear and long-range dependencies that are common in real-world traffic systems. With the rise of deep learning, sequence-based models such as Recurrent Neural Networks (RNNs) [8], Long Short-Term Memory (LSTM) [9], and Gated Recurrent Units (GRUs) [10] have been widely used to model temporal dependencies. Although these models improve forecasting accuracy, their sequential nature limits parallelization and often results in inefficient training. Additionally, these models often suffer from gradient vanishing and instability when processing long temporal sequences. To mitigate these limitations, convolution-based architectures have been introduced for temporal modeling. Temporal Convolutional Networks (TCNs) [11] leverage causal and dilated convolutions to capture long-range temporal dependencies while preserving causality and supporting parallel computation. Meanwhile, Transformer architectures have also been applied to time-series forecasting because of their strong ability to capture global dependencies [12,13]. However, their performance in traffic forecasting remains unstable due to data heterogeneity and high computational cost. In terms of spatial modeling, early deep learning approaches applied Convolutional Neural Networks (CNNs) to grid-based traffic maps, but such Euclidean designs cannot represent the irregular and directional structures of real road networks [14]. Graph Convolutional Networks (GCNs) [15,16] have emerged as the dominant paradigm for capturing spatial dependencies on irregular graph structures. Models such as STGCN [17] and DCRNN [18] combined graph convolution with temporal modeling to capture joint spatio-temporal features, while AGCRN [19] further introduced node-level embeddings to handle heterogeneous regions. More recently, attention-based spatial models such as GMAN [20] and STGAT [21] have used Transformer-style attention to dynamically learn node relationships without fixed adjacency matrices. These models achieve higher flexibility and accuracy but incur heavy computational cost due to dense attention, which limits scalability on large traffic networks.

Despite these advancements, real-world traffic forecasting still faces several core challenges. First, many spatial encoders rely on fixed graph topologies or compute dense attention matrices across all nodes. Fixed adjacency matrices are often derived from static road structures and fail to reflect real-time traffic dynamics or varying node relevance. In contrast, dense attention mechanisms introduce quadratic computational costs, hindering scalability for large-scale networks. Second, temporal modeling remains computationally demanding. Although strong locality and periodicity exist in traffic time series, many models still rely on complex recurrent or full-attention architectures, resulting in high training cost and limited efficiency. Finally, most existing graph-based methods also overlook directional and multi-hop diffusion behaviors that characterize real traffic propagation. This lack of directional awareness hinders the accurate modeling of asymmetric and long-range interactions, such as congestion ripple effects spreading along interconnected road segments.

The aforementioned challenges highlight the need for a unified and efficient spatio-temporal modeling paradigm that can jointly handle temporal evolution and dynamic spatial dependencies within complex road networks. To this end, we propose DSSA-TCN, a novel architecture that integrates adaptive sparse spatial attention and bidirectional diffusion graph convolution within a causal temporal convolutional backbone. Unlike previous hybrid frameworks such as Graph WaveNet, MegaCRN, and STAEformer, which loosely combine graph or attention modules, DSSA-TCN redefines spatio-temporal learning as a layer-wise alternating and tightly coupled process, enabling temporal and spatial representations to co-evolve within each block. Each DSSA-TCN block consists of three key components: temporal convolution, adaptive sparse attention, and diffusion graph convolution. The temporal module employs dilated causal convolutions to efficiently capture long-range dependencies while preserving causality and parallel computation. The adaptive sparse attention mechanism dynamically selects the most informative neighbors for each node, reducing spatial complexity from *O*(*N*^2^) to *O*(*kN*) while maintaining essential structural correlations. Following attention, the diffusion convolution operates over the complete directed topology to model directional and multi-hop propagation, ensuring that spatial reasoning remains both physically interpretable and topologically consistent. This alternating spatio-temporal coupling allows DSSA-TCN to achieve a balance between accuracy, interpretability and scalability. By aligning adaptive sparsity with topology-aware diffusion, the model effectively captures both global context and localized directional flow, achieving a compact yet expressive form of spatio-temporal reasoning. Such a design ensures that information propagation across layers is jointly regulated by temporal causality and spatial adaptivity, resulting in improved efficiency and stability.

In summary, DSSA-TCN unifies adaptive sparse attention and diffusion-based spatial reasoning within a lightweight temporal backbone, providing a new paradigm for scalable and interpretable traffic forecasting. Extensive experiments on six real-world traffic datasets demonstrate that DSSA-TCN achieves superior predictive accuracy and computational efficiency, validating the effectiveness of its layer-wise coupling mechanism.

The main contributions of this paper are summarized as follows:

- We propose DSSA-TCN, a unified deep learning framework that unifies adaptive sparse attention and bidirectional diffusion graph convolution within a causal temporal backbone, enabling tightly coupled spatio-temporal modeling and efficient information propagation.
- We design an adaptive sparse attention module that dynamically selects high-impact nodes, reducing spatial complexity from *O*(*N*^2^) to *O*(*kN*) while preserving key structural dependencies. In parallel, a bidirectional multi-order diffusion process captures directional and multi-hop propagation, improving interpretability and scalability.
- We conduct extensive experiments on six real-world traffic datasets, showing that DSSA-TCN consistently surpasses state-of-the-art baselines in accuracy and efficiency, with strong robustness on large graphs and long-term forecasting tasks.

## Related work

### Traffic flow forecasting

Traffic flow forecasting remains a fundamental task in intelligent transportation systems, aiming to predict future traffic states from historical observations. The problem is inherently complex due to nonlinear temporal dynamics, periodic variations, and intricate spatial dependencies shaped by large-scale and irregular road networks. In practice, effective forecasting models must also achieve a balance between accuracy, computational efficiency, and scalability for real-time urban operations. Early-stage approaches such as ARIMA and SVR [22,23] provided interpretable and lightweight solutions but relied on strong linear and stationary assumptions. With the emergence of deep learning, recurrent neural networks (RNNs), long short-term memory (LSTM), and gated recurrent units (GRUs) [24–26] significantly improved temporal sequence modeling. However, their inherently sequential structure restricts parallel computation and struggles to capture long-range temporal dependencies, which limits their efficiency in large-scale forecasting tasks. To overcome these limitations, convolutional architectures were subsequently introduced to enable parallel temporal modeling. Models such as DeepST [27] and ST-ResNet [28] applied temporal and spatial convolutions separately to extract local and periodic patterns, demonstrating strong short-term performance. Nonetheless, their grid-based Euclidean assumptions hindered their ability to represent the non-Euclidean topology of real traffic networks. Later designs such as STNN [29] combined convolution with attention mechanisms to enhance spatial–temporal fusion, but at the cost of higher architectural complexity. Building upon these foundations, hybrid and graph-enhanced frameworks emerged to jointly capture spatial and temporal dependencies. T-GCN [25] integrates graph convolution with GRU for dynamic spatio-temporal learning, while SRCN [30] leverages residual convolutions for end-to-end city-scale prediction. In addition, normalization-based models like ST-Norm [31] decouple spatial and temporal variations, improving stability and convergence. These efforts collectively mark the transition from purely temporal or grid-based designs to more unified spatio-temporal representations.

Collectively, these developments have paved the way for more advanced modeling paradigms that aim to better capture spatial complexity and temporal heterogeneity. In particular, recent research trends have focused on structured spatial reasoning using graph-based models and global dependency modeling via attention-based architectures. These directions will be discussed in detail in the following sections.

### Graph convolution network

Graph Convolutional Networks (GCNs) have become the cornerstone of spatial modeling in non-Euclidean traffic networks, offering a principled framework for capturing interactions among irregularly connected sensors and road segments [32]. Early GCN-based approaches, such as DCRNN [18] and STGCN [17], pioneered the integration of diffusion graph convolution and temporal convolution to jointly model spatio-temporal dependencies. However, their reliance on static and predefined adjacency matrices limits adaptability to dynamic traffic conditions and context-dependent relationships. To address this limitation, subsequent models introduced adaptive or data-driven graph structures. Graph WaveNet [33] proposed a learnable adjacency matrix to decouple spatial dependencies from fixed topology, while AGCRN [34] incorporated node-specific embeddings to capture regional heterogeneity. More recently, DSTAGNN [35] extended this paradigm by dynamically updating graph connections according to evolving traffic contexts, enabling fine-grained adaptation to temporal variations. Similarly, FC-STGNN [36] explored fully connected graph structures to capture global correlations, though such approaches inevitably increase computational overhead in large-scale networks. With these advancements, dynamic and diffusion-based frameworks have further improved physical interpretability and multi-scale spatial reasoning. DGCRN [37] learns time-varying graphs conditioned on evolving traffic contexts, while MegaCRN [38] employs a meta-graph learning strategy to dynamically generate latent adjacency matrices. MTGNN [39] integrates multi-hop diffusion to model directional propagation across the network, balancing local influence and global context. STGNN-TCN [40] further couples temporal convolution with graph diffusion, demonstrating the potential of hybrid architectures for scalable and interpretable spatio-temporal learning.

Despite these developments, GCN-based frameworks continue to face challenges in achieving a balance between adaptability and computational efficiency, as well as in maintaining consistency between spatial diffusion and temporal modeling. Future research should focus on developing unified, physically interpretable spatio-temporal frameworks that integrate diffusion-based spatial reasoning with temporally coherent learning.

### Transformer

Transformer-based architectures have recently emerged as a powerful paradigm for spatio-temporal modeling, owing to their ability to capture long-range dependencies and global contextual relationships. This capability has made them increasingly popular in traffic forecasting, where complex and interdependent dynamics must be modeled efficiently across both temporal and spatial domains. Early efforts sought to integrate the attention mechanism into existing spatio-temporal frameworks. For instance, ASTGCN [41] introduced spatial and temporal attention modules into graph-based networks, while GMAN [20] employs multi-head spatio-temporal attention to learn latent graphs at each time step. STAEformer [42] further introduces spatio-temporal adaptive embeddings within an encoder–decoder architecture to better capture periodic and fluctuating patterns. More recent approaches, such as HUTFormer [43], design hierarchical U-shaped Transformer structures to model multi-scale dependencies, whereas GATFormer [44] integrates graph structural priors into Transformer encoders to improve topology awareness. Despite these advances, Transformer-based models often face intrinsic challenges when applied to traffic networks. Their dense attention mechanisms compute all-to-all interactions across nodes or time steps, resulting in quadratic complexity and redundant dependency learning. STPFormer [45] introduces pivot-node-based sparse attention to mitigate the quadratic complexity of dense attention while preserving key spatio-temporal dependencies. In parallel, other domains have explored context-aware spatio-temporal reasoning under attention-driven paradigms. For instance, Chen *et al*. [46] proposed a multi-context aware human mobility prediction framework that integrates motif-preserving hypergraph convolution and social-gated fusion to capture higher-order mobility motifs and contextual dependencies. Although focused on individual mobility behavior rather than large-scale traffic flow, this work highlights how incorporating structured priors and multi-context attention can enhance the interpretability and generalization of spatio-temporal models.

Building on these insights, recent Transformer-based research continues to investigate structured and sparse attention mechanisms as a means to enhance scalability while maintaining essential dependencies. Sparse attention selectively emphasizes the most relevant spatial or temporal relationships, thereby reducing redundant computations and highlighting dominant propagation patterns within complex networks. However, the adaptive and topology-aware integration of such sparsity remains insufficiently explored, leaving room for more unified and physically interpretable modeling frameworks in future work.

## Materials

In this section, we formally define the problem of traffic flow forecasting, including the modeling of the traffic network as a graph, the traffic signal definition, and the formulation of the forecasting task. We also describe how the topology graph is constructed from spatial relationships.

**Traffic network and topology graph.** We represent the transportation system as a directed graph G=(V,E), where *V* is the set of N=|V| nodes, each corresponding to a traffic sensor or road junction, and *E* is the set of edges representing directional relationships between nodes. These spatial relationships can be constructed based on real-world road connectivity or computed from physical distances between nodes. Specifically, the topology graph is represented by an adjacency matrix $𝐴\in {\mathbb{R}}^{N\times N}$, where the entry $A_{ij}$ indicates the influence from node *i* to node *j*. In our setting, the adjacency matrix is derived from a spatial distance matrix $𝐷\in {\mathbb{R}}^{N\times N}$, where each element $D_{ij}$ denotes the Euclidean or geodesic distance between node $V_{i}$ and $V_{j}$. The binary connectivity or weighted edge value is then computed as:

$$A_{ij}=\left\{ \begin{array}{cc} \exp\left(-\frac{D_{ij}}{\sigma^{2}}\right), & \text{ if }D_{ij}<\tau \\ 0, & \text{ otherwise } \end{array}\right.$$ (1)

where *σ* is a scaling factor and *τ* is a distance threshold that controls the sparsity of the graph. This diffusion-based adjacency matrix enables the modeling of local proximity while preserving physical topology structure.

**Traffic signal representation.** Let denote the traffic signal at time $X_{t}\in {\mathbb{R}}^{N\times C}$ denote the traffic signal at time *t*, where *C* is the number of features recorded by each sensor (e.g., traffic volume, speed, time-of-day, day-of-week). Given a historical window of *T*_*h*_ time steps, the spatio-temporal input sequence is defined as:

$${𝒳}_{t}=\left\{X_{t-T_{h}+1},X_{t-T_{h}+2},\ldots ,X_{t}\right\}\in {\mathbb{R}}^{T_{h}\times N\times C}$$ (2)

**Problem definition.** Given the historical traffic signals ${𝒳}_{t}$ and the graph topology *A*, the goal is to learn a predictive function f(·) that estimates future traffic states over the next *T*_*f*_ time steps:

$${\hat{𝒴}}_{t}=f\left({𝒳}_{t},A\right)\in {\mathbb{R}}^{T_{f}\times N\times 1}$$ (3)

This function should effectively capture the spatio-temporal dependencies across both sensor locations and time, enabling accurate multi-step forecasting for all nodes in the network.

## Methodology

### Overall framework

The proposed DSSA-TCN model is designed to effectively capture spatio-temporal dependencies in traffic networks by integrating three key components: a temporal convolutional network for long-range temporal modeling, an adaptive sparse attention mechanism for dynamic spatial interaction, and a diffusion-based graph convolution for directional and multi-hop propagation. Given an input sequence ${𝒳}_{t}\in {\mathbb{R}}^{T_{h}\times N\times C}$, where *T*_*h*_ is the number of historical time steps, *N* is the number of traffic sensors, and *C* is the number of features per node, the goal is to predict future traffic states over the next *T*_*f*_ steps, denoted by ${\hat{𝒴}}_{t}\in {\mathbb{R}}^{T_{f}\times N\times 1}$, The underlying spatial structure is encoded in an adjacency matrix $𝐴\in {\mathbb{R}}^{N\times N}$, which reflects the connectivity of the traffic network.

**Fig 1:**
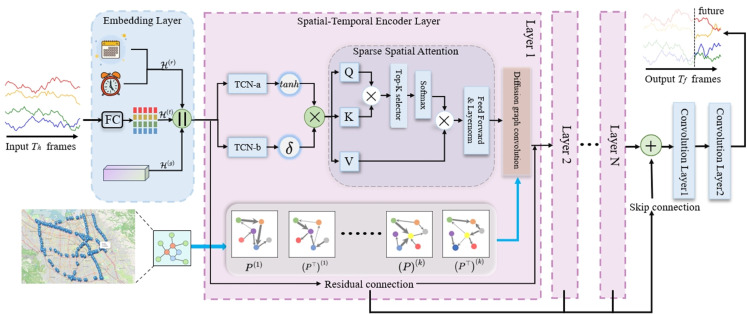
The overall architecture of DSSA-TCN.

As shown in Fig 1, the model first transforms the raw inputs into a latent representation through a linear projection, and augments it with time-of-day, day-of-week, and learnable node embeddings. These embeddings help the model capture periodic traffic patterns and spatial heterogeneity. Temporal modeling is performed using stacked layers of dilated causal convolutions applied along the time axis. The causal structure ensures that predictions are made using only past information, while dilation enlarges the temporal receptive field without increasing model depth. Compared to recurrent networks, this structure enables greater parallelism and improved training efficiency. To capture spatial dependencies, each temporal block is followed by a spatial modeling module consisting of two parts. First, an adaptive attention mechanism computes relevance scores across nodes and selects a sparse subset of top-ranked neighbors for each target node. This reduces unnecessary computation and enables the model to focus on structurally meaningful connections. Then, the attended features are passed through a diffusion convolutional layer, which aggregates information from multi-hop neighbors along both forward and reverse directions on the graph. Outputs from multiple blocks are connected through skip connections to preserve multi-scale representations. After stacking all layers, a lightweight convolutional head is applied to produce the final predictions over all nodes and time steps. By alternating between temporal and spatial modules, DSSA-TCN is able to jointly learn temporally coherent and spatially structured representations, resulting in accurate and efficient traffic forecasting on complex road networks.

### Input embedding layer

To enhance the model’s ability to capture complex spatio-temporal dependencies, we construct an input embedding layer that encodes raw traffic observations into a unified latent representation. This layer integrates three complementary components: raw feature projection, temporal context embedding, and learnable node embedding. Together, they enrich the input tensor with temporal periodicity and spatial semantics, facilitating effective representation learning for traffic forecasting.

**Raw feature projection.** The input traffic sequence $𝒳\in {\mathbb{R}}^{T_{h}\times N\times C}$ includes real-valued input features such as traffic volume or speed. A fully connected linear layer is applied to project these features into a hidden representation space of dimension *d*_*r*_, producing ${ℋ}^{\left(r\right)}\in {\mathbb{R}}^{T_{h}\times N\times d_{r}}$. This transformation allows the model to extract more expressive patterns from the original input, providing a stronger foundation for downstream processing.

**Temporal context embedding.** Given the periodic nature of urban traffic, we introduce time-aware embeddings to capture short-term and long-term temporal patterns.

- Time-of-Day (ToD): Each timestamp is discretized into one of *P* intervals throughout the day (e.g., 288 intervals representing 5-minute steps), and mapped into a latent vector using a learnable embedding matrix of size $P\times d_{t}$;
- Day-of-Week (DoW): Each day is encoded as one of seven categorical indices and mapped using an embedding matrix of size $7\times d_{t}$;

The resulting embeddings are broadcasted across all nodes at each time step and concatenated into a joint temporal context embedding ${ℋ}^{\left(t\right)}\in {\mathbb{R}}^{T_{h}\times N\times 2d_{t}}$. These representations help the model to distinguish between different traffic regimes, such as weekday peak hours versus weekend off-peak periods.

**Learnable node embedding.** To account for spatial heterogeneity across traffic sensors, each node is assigned a trainable node-specific embedding vector. These embeddings ${ℰ}_{\text{node }}\in {\mathbb{R}}^{N\times d_{g}}$ are shared across time and replicated over the temporal dimension to obtain ${ℋ}^{\left(g\right)}\in {\mathbb{R}}^{T_{h}\times N\times d_{g}}$. By incorporating these node-specific representations, the model gains the ability to differentiate sensor behaviors and learn spatial biases adaptively, which is particularly beneficial in dynamically changing traffic environments.

Finally, all three components are concatenated along the feature axis to form the final input embedding:

$${ℋ}_{\text{embed }}=\left[{ℋ}^{\left(r\right)}\left\|{ℋ}^{\left(t\right)}\right\|{ℋ}^{\left(g\right)}\right]\in {\mathbb{R}}^{T_{h}\times N\times d_{e}}$$ (4)

where $d_{e}=d_{r}+2d_{t}+d_{g}$ is the total embedding dimension.

### Spatial-temporal encoder layer

#### Temporal correlation capturing.

To effectively capture temporal dependencies in traffic data, we employ a gated dilated causal convolution module as the temporal encoder in our model. Compared to recurrent architectures, this structure enables parallel computation, avoids gradient vanishing, and preserves strict time causality by constraining each output to depend solely on past inputs. Let the initial embedded input be denoted as ${ℋ}_{\text{embed }}\in {\mathbb{R}}^{T\times N\times d_{e}}$. Before feeding into the temporal encoder, the embedding tensor is first permuted to the shape ${\mathbb{R}}^{d_{e}\times N\times T}$, so that a temporal 1D convolution can be applied along the time axis. A 1×1 convolution is then used to project the feature dimension from $d_{e}$ to $d_{h}$, resulting in ${ℋ}^{\left(0\right)}\in {\mathbb{R}}^{d_{h}\times N\times T}$. This transformed representation serves as the input to the first temporal block. For each layer l∈{0,1,…,L − 1}, the input ${ℋ}^{\left(l\right)}\in {\mathbb{R}}^{d_{h}\times N\times T}$ is processed by the module to produce ${\tilde{ℋ}}^{\left(l\right)}$, which will subsequently be fed into the spatial modeling unit of the same layer. The module operation is defined as:

$${\tilde{ℋ}}^{\left(l\right)}=\tanh\left(\theta_{1}^{\left(l\right)}⋆{ℋ}^{\left(l\right)}+b^{\left(l\right)}\right)⊙\sigma \left(\theta_{2}^{\left(l\right)}⋆{ℋ}^{\left(l\right)}+c^{\left(l\right)}\right)$$ (5)

Here, ⋆ denotes a dilated causal convolution, ⊙ represents element-wise multiplication, and tanh(·), σ(·) are the hyperbolic tangent and sigmoid activation functions. The learnable parameters include the convolutional kernels $\theta_{1}^{\left(l\right)},\theta_{2}^{\left(l\right)}\in {\mathbb{R}}^{d_{h}\times d_{h}\times k},$ and biases $b^{\left(l\right)},c^{\left(l\right)}\in {\mathbb{R}}^{d_{h}}$, where 𝑘 is the kernel size.

The dilated convolution operation for a filter $\theta_{p}\in {\mathbb{R}}^{d_{h}\times d_{h}\times k}$ is given by:

$$\left[\theta_{p}⋆{ℋ}^{\left(l\right)}\right](t)=\sum_{s=1}^{M}\theta_{p}(s)\cdot {ℋ}^{\left(l\right)}(t-d\cdot s)$$ (6)

where *d* is the dilation factor, which increases exponentially across layers (e.g., d=1,2,4,…). This design allows the model to expand the receptive field exponentially without deepening the network, making long-range temporal dependencies easier to learn.

By integrating the filter and gate branches through a gated activation mechanism, the model is able to dynamically regulate temporal information flow, retaining informative patterns while suppressing noise. The output of each layer is forwarded to the spatial modeling unit, enabling the network to alternately learn temporal and spatial structures in a unified framework.

#### Sparse spatial attention.

To capture dynamic and fine-grained spatial dependencies among traffic sensors, we introduce a sparse spatial attention (SSA) mechanism following each temporal convolutional layer. Unlike standard attention mechanisms methods that compute interactions between all node pairs indiscriminately, our method selectively attends to a subset of the most relevant nodes per target, thereby reducing computational overhead and improving robustness against noisy or irrelevant dependencies.

Given the output of the *l* temporal convolutional block ${\tilde{ℋ}}^{\left(l\right)}\in {\mathbb{R}}^{d_{h}\times N\times T}$, we first permute it to ${\mathbb{R}}^{T\times N\times d_{h}}$, and then project it into query, key, and value representations through learnable linear transformations:

$$𝐐={𝐖}_{Q}{\tilde{ℋ}}^{\left(l\right)},\;𝐊={𝐖}_{K}{\tilde{ℋ}}^{\left(l\right)},\;𝐕={𝐖}_{V}{\tilde{ℋ}}^{\left(l\right)}$$ (7)

where ${𝐖}_{Q},{𝐖}_{K},{𝐖}_{V}\in {\mathbb{R}}^{d_{h}\times d^{\prime }}$ are the trainable parameters, and $d^{\prime }$ is the attention dimension. We compute pairwise attention scores among all nodes using scaled dot-product attention:

$$𝐀=\mathrm{Softmax}\left(\frac{𝐐{𝐊}^{⊤}}{\sqrt{d^{\prime }}}\right)$$ (8)

The Softmax operation normalizes the pairwise similarity scores into a probability distribution, ensuring that all attention weights are positive and sum to one for each target node. This normalization allows the model to interpret the relative importance of neighboring nodes and stabilizes the gradient updates during training.

To promote sparsity and interpretability, we apply an adaptive *Top*–*k* filtering strategy. Specifically, a learnable scalar *α* is used to dynamically determine the number of top-attended neighbors per node:

$$k=\max\left(1,\left\lfloor N\cdot \left(\lambda_{\min}+\left(\lambda_{\max}-\lambda_{\min}\right)\cdot \sigma (\alpha )\right)\right\rfloor \right)$$ (9)

where $\lambda_{\min},\lambda_{\max}\in (0,1)$ are predefined lower and upper sparsity bounds, and σ(·) is the sigmoid function. For each row of **A**, we retain only the *Top*–*k* values are retained while the rest are masked with −∞, followed by a second softmax normalization to ensure valid attention weights. This process yields the sparse attention matrix ${𝐀}_{\text{top }-k}$.

The final sparse spatial representation is computed by aggregating the value vectors through the filtered attention weights:

$${𝒵}^{\left(l\right)}={𝐀}_{\text{top }-k}\cdot 𝐕$$ (10)

This attention mechanism allows the model to automatically select the most informative spatial interactions per node at each time step, effectively filtering out irrelevant signals while preserving key structural dependencies. The resulting sparse representation ${𝒵}^{\left(l\right)}\in {\mathbb{R}}^{T\times N\times d_{h}}$ is then passed to the diffusion graph convolution module for further spatial modeling.

#### Diffusion graph convolution.

To further capture the directional and multi-hop spatial dependencies in traffic networks, we incorporate a diffusion graph convolution (DGC) module into the model. This module performs feature propagation over a static graph topology, allowing the network to learn structured spatial representations based on physical connectivity.

Let $A\in {\mathbb{R}}^{N\times N}$ be the predefined adjacency matrix of the traffic network, representing the connectivity among sensors. To enhance self-awareness in the diffusion process, we add self-loops and construct the one-step random walk transition matrix as:

$$\hat{𝐀}=𝐀+𝐈,\;P={𝐃}^{-1}\hat{𝐀}$$ (11)

where **I** is the identity matrix and **D** is the diagonal out-degree matrix of $\hat{𝐀}$. Here, *P* represents the one-step transition probability between connected sensors, describing how traffic information from a node is distributed to its immediate neighbors. The self-loop term ensures that each node retains part of its own signal, avoiding feature dilution. The 𝑘-step diffusion process is then defined as:

$$P_{ij}^{\left(k\right)}={\left(\frac{{\hat{A}}_{ij}}{\sum_{j}{\hat{A}}_{ij}}\right)}^{k}$$ (12)

Intuitively, *P*${\;}^{\left(\text{k}\right)}$ quantifies how strongly node *j* influences node *i* after *k* successive propagation steps, thereby encoding multi-hop spatial dependencies across the traffic graph. Let ${𝒵}^{\left(l\right)}\in {\mathbb{R}}^{T\times N\times d_{h}}$ be the input representation at layer *l*, produced by the preceding sparse attention module. The diffusion graph convolution aggregates spatial information from multiple hops through linear projections over the diffusion matrices:

$${𝒢}^{\left(l\right)}=\sum_{k=0}^{K}P^{\left(k\right)}\cdot {𝒵}^{\left(l\right)}\cdot {𝐖}_{k}$$ (13)

where ${𝐖}_{k}\in {\mathbb{R}}^{d_{h}\times d_{h}}$ is a trainable weight matrix for the 𝑘-th diffusion order, and 𝐾 is the maximum number of hops. This formulation can be viewed as a weighted combination of signals diffused through 0-hop (self), 1-hop, ... , *K*-hop neighbors, where each term *P*${\;}^{\left(\text{k}\right)}$ models a distinct receptive field and ${𝐖}_{k}$ learns its importance. In essence, it allows each node to integrate both local and distant contextual information.

In traffic systems, vehicles move along directed roads, where information propagates from upstream to downstream and can also feed back upstream; employing both *P* and $P^{⊤}$ enables the model to more faithfully represent this asymmetric bidirectional interaction. The resulting spatial representation ${𝒢}^{\left(l\right)}\in {\mathbb{R}}^{T\times N\times d_{h}}$ is then passed to the subsequent temporal modeling layer or the final output projection module.

### Output projection layer

To generate the final traffic predictions, we design an output projection layer that aggregates the deep spatio-temporal representations from all preceding blocks and maps them into the target prediction space. This module performs two key functions: multi-scale feature fusion and step-wise forecasting. Let the output of the 𝑙-th spatio-temporal block be denoted as ${𝒢}^{\left(l\right)}\in {\mathbb{R}}^{d_{h}\times N\times T}$ is the number of time steps. To integrate hierarchical temporal-spatial features, we apply a skip connection mechanism by projecting each block’s output into a unified feature space of dimension *f*, and aggregating them as follows:

$$𝒮=\sum_{l=1}^{L}{𝐖}_{s}^{\left(l\right)}\cdot {𝒢}^{\left(l\right)}$$ (14)

where ${𝐖}_{s}^{\left(l\right)}\in {\mathbb{R}}^{d_{h}\times 1}$ is a learnable linear projection matrix for the *l*-th block, and the fused skip representation satisfies $𝒮\in {\mathbb{R}}^{d_{h}\times N\times 1}$.

We then apply a lightweight prediction head consisting of two linear layers with a nonlinear activation in between to generate the final multi-step predictions:

$${\hat{𝒴}}_{t}={𝐖}_{2}\cdot \sigma \left({𝐖}_{1}\cdot 𝒮\right)\in {\mathbb{R}}^{T_{f}\times N\times 1}$$ (15)

Here, ${𝐖}_{1}\in {\mathbb{R}}^{d_{e}\times d_{h}}$ and ${𝐖}_{2}\in {\mathbb{R}}^{T_{f}\times d_{e}}$ are trainable weight matrices, $d_{e}$ is the intermediate embedding dimension, and σ(·) denotes a nonlinear activation function (e.g., ReLU). This output projection layer enables the model to decode high-level spatio-temporal features into fine-grained, time-aligned predictions for each sensor over the forecast horizon.

### Loss function

To train the model for accurate multi-step traffic flow forecasting, we adopt the mean absolute error (MAE) as the optimization objective. MAE is robust to outliers, numerically stable, and widely used in temporal regression tasks such as traffic prediction.

Let the model’s predicted output be ${\hat{𝒴}}_{t}\in {\mathbb{R}}^{T_{h}\times N\times 1}$, and the corresponding ground truth be ${𝒴}_{t}\in {\mathbb{R}}^{T_{h}\times N\times 1}$. The overall loss function is defined as:

$$ℒ=\frac{1}{T_{h}N}\sum_{t=1}^{T_{f}}\sum_{n=1}^{N}\left|{\hat{𝒴}}_{t}(t,n)-{𝒴}_{t}(t,n)\right|$$ (16)

where *T*_*f*_ denotes the prediction horizon and *N* is the number of traffic sensor nodes. ${\hat{𝒴}}_{t}(t,n)$ and ${𝒴}_{t}(t,n)$ represent the predicted and ground truth values for node *n* at future time step *t*. This loss function effectively quantifies the model’s forecasting accuracy across temporal and spatial dimensions, guiding parameter optimization during training to improve predictive performance.

## Experiments

### Experimental setup

**Datasets.** To thoroughly evaluate the generalization and robustness of the proposed DSSA-TCN model, we conduct experiments on six widely-used benchmark datasets: METR-LA, PEMS-BAY, PEMS03, PEMS04, PEMS07, and PEMS08. These datasets differ significantly in terms of geographic coverage, spatial scale, and traffic modalities, providing a comprehensive testbed for spatio-temporal forecasting. Specifically, METR-LA and PEMS-BAY are traffic speed datasets published by Li *et al*. [18], containing 5-minute interval measurements collected from 207 and 325 sensors in Los Angeles and the Bay Area, respectively. Both datasets are divided into training (70%), validation (10%), and testing (20%) splits. The remaining four datasets, PEMS03, PEMS04, PEMS07, and PEMS08, are sourced from the caltrans performance measurement system (PeMS) and introduced by Zheng *et al*. [20]. These datasets record traffic flow at 5-minute intervals from sensors deployed in various Caltrans districts. They are split into 60% training, 20% validation, and 20% testing sets. All data are preprocessed using z-score normalization based on the statistics of the training set. A summary of dataset statistics is provided in Table 1. Notably, METR-LA and PEMS-BAY are used for traffic speed prediction, while the other four datasets are used for traffic flow forecasting.

**Table 1:** Summary of benchmark datasets.

Dataset	#Nodes	Time Interval	#Timesteps	Start Time	End Time
METR-LA	207	5 minutes	34,272	03/2012	06/2012
PEMS-BAY	325	5 minutes	52,116	01/2017	05/2017
PEMS03	358	5 minutes	26,209	05/2012	07/2012
PEMS04	307	5 minutes	16,992	01/2018	02/2018
PEMS07	883	5 minutes	28,224	05/2017	08/2017
PEMS08	170	5 minutes	17,856	07/2016	08/2016

**Settings.** All experiments are conducted on a Linux server equipped with an Intel i9-10900K CPU and an NVIDIA RTX 4080 GPU. The model is implemented using PyTorch 2.3.0 with CUDA 12.1 support. Optimization is performed using the Adam optimizer with an initial learning rate of 0.002 and a weight decay of 0.0003. A multi-step learning rate scheduler decays the learning rate by a factor of 0.1 at the 50th and 70th epochs. Each model is trained for 100 epochs with a batch size of 64, except for PEMS07, where a batch size of 8 is used due to its larger graph scale. The training objective is masked mean absolute error (MAE), and all results are averaged over three independent runs to reduce variance.

Each node’s input series is projected into a 12-dimensional embedding space. To capture temporal and spatial heterogeneity, three additional 12-dimensional embeddings are concatenated: time-of-day, day-of-week and a learnable node embedding. The temporal modeling backbone consists of four TCN blocks, each with two layers of gated dilated convolutions (kernel size = 2). Both the residual and dilation branches use 32 channels. Skip connections and the final projection layer use 256 and 512 channels respectively. Dropout is applied with a fixed rate of 0.3 for regularization. For spatial modeling, we employ a one-layer sparse multi-head self-attention module with 4 attention heads and a hidden feedforward dimension of 128. An adaptive top-*k* selection mechanism dynamically selects the most relevant neighbors, where the *k* is constrained within the range of 1/6 to 1/3 of the total nodes. Following the attention operation, a bidirectional diffusion graph convolution is applied using a diffusion order of 2, allowing information to propagate through both forward and reverse 2-hop neighbors based on the normalized adjacency matrices.

**Metrics.** To comprehensively evaluate the predictive performance of our model, we adopt three widely-used evaluation metrics: mean absolute error (MAE), mean absolute percentage error (MAPE), and root mean squared error (RMSE). These metrics are defined as follows:

- **MAE** measures the average absolute deviation between predicted and ground truth values:
  $$MAE=\frac{1}{\left|𝒴\right|}\sum_{\left(i,t\right)}\left|{\hat{y}}_{i,t}-y_{i,t}\right|$$ (17)
- **MAPE** quantifies the average relative error in percentage terms:
  $$\text{ MAPE }=\frac{1}{\left|𝒴\right|}\sum_{\left(i,t\right)}\left|\frac{{\hat{y}}_{i,t}-y_{i,t}}{y_{i,t}}\right|\times 100\%$$ (18)
- **RMSE** penalizes larger errors more heavily by computing the square root of the mean squared error:
  $$RMSE=\sqrt{\frac{1}{\left|𝒴\right|}\sum_{\left(i,t\right)}{\left({\hat{y}}_{i,t}-y_{i,t}\right)}^{2}}$$ (19)

where ${\hat{y}}_{i,t}$ and *y*_*i*,*t*_ denote the predicted and ground truth values for node *i* at time *t*, and |𝒴| is the total number of valid prediction points. All metrics are reported under three common forecasting horizons: 3-step (15 minutes), 6-step (30 minutes), and 12-step (60 minutes), enabling a comprehensive evaluation of model performance across short-term, medium-term, and long-term intervals.

### Experiment result

To thoroughly evaluate the effectiveness and generalization ability of our proposed DSSA-TCN, we compare it against six representative baselines spanning a diverse set of spatio-temporal forecasting paradigms. All models are implemented or re-implemented within the BasicTS [47] framework to ensure consistency in data preprocessing, training protocols, and evaluation metrics.

- **DCRNN** [18]: A pioneering model that integrates recurrent neural networks with bidirectional diffusion graph convolutions. It captures temporal dynamics through an encoder-decoder architecture based on GRUs, while modeling asymmetric spatial dependencies via directed diffusion processes;
- **STGCN** [17]: A fully convolutional framework that alternates between temporal convolutions and spatial GCN layers. By eliminating recurrent structures, it improves training efficiency while synchronously modeling spatial and temporal patterns;
- **Graph WaveNet** [33]: Enhances temporal modeling with dilated causal convolutions and spatial adaptivity via a learnable adjacency matrix. This design improves robustness to noisy or incomplete graph structures and supports long-range dependency learning;
- **ST-Norm** [31]: Introduces spatial-temporal normalization modules that standardize node-level sequences using local statistics. It mitigates distributional heterogeneity across nodes and time, leading to improved generalization across varying traffic regimes;
- **MegaCRN** [38]: Employs a meta-graph convolution module to dynamically construct instance-specific graph structures during training. This allows the model to capture time-varying spatial correlations without relying on static or predefined topologies;
- **STAEformer** [42]: A Transformer-based architecture that introduces spatio-temporal adaptive embeddings to model periodic patterns and spatial heterogeneity.

These baselines span multiple modeling paradigms, including sequence-based RNNs, convolutional encoders, graph neural networks with adaptive structures, and Transformer-based architectures. Together, they provide a comprehensive and rigorous benchmark suite for validating the performance and generalization ability of DSSA-TCN.

We conducted a comprehensive comparison between our proposed DSSA-TCN model and six representative spatio-temporal forecasting baselines across six real-world traffic datasets: METR-LA, PEMS-BAY, PEMS03, PEMS04, PEMS07, and PEMS08. On the METR-LA and PEMS-BAY datasets, we further evaluated model performance under multiple prediction horizons, specifically 15, 30, and 60 minutes, as reported in Tables 2 and 3. Overall, DSSA-TCN achieved the best results in most experimental settings. According to Table 2, DSSA-TCN ranked first in 10 out of 18 metric-dataset combinations. The advantages were particularly evident on datasets with complex traffic dynamics such as PEMS-BAY, PEMS04, and PEMS08. For example, on the PEMS-BAY dataset, DSSA-TCN achieved MAE of 1.53, RMSE of 3.50, and MAPE of 3.42%, outperforming all baseline models. On PEMS04, it also achieved the lowest RMSE (30.51) and MAPE (12.46%), significantly surpassing competitive models like MegaCRN and ST-Norm.

**Table 2:** Overall performance comparison on six benchmark traffic datasets.

Dataset	METR-LA			PEMS-BAY			PEMS03			PEMS04			PEMS07			PEMS08			1${\;}^{\text{st}}$ Count
Metric	MAE	RMSE	MAPE	MAE	RMSE	MAPE	MAE	RMSE	MAPE	MAE	RMSE	MAPE	MAE	RMSE	MAPE	MAE	RMSE	MAPE	
**DCRNN**	3.04	6.28	8.31%	1.60	3.72	3.64%	15.60	27.61	15.43%	19.67	31.18	13.64%	24.31	37.21	9.71%	15.37	24.21	10.31%	0
**STGCN**	3.14	6.33	8.46%	1.69	3.79	3.84%	15.84	27.99	15.48%	19.91	31.57	13.75%	22.23	35.78	9.54%	16.48	25.63	11.07%	0
**GWNet**	3.03	6.17	8.23%	1.61	3.77	3.58%	**14.52**	**25.40**	15.45%	18.70	30.53	13.07%	20.13	32.94	8.88%	14.61	**23.47**	9.61%	3
**ST-Norm**	3.13	6.43	8.62%	1.59	3.69	3.66%	15.24	26.36	15.55%	19.12	31.98	12.85%	20.51	34.46	8.67%	15.36	25.03	9.71%	0
**MegaCRN**	2.96	6.05	8.18%	1.54	3.58	3.45%	14.91	25.96	15.50%	18.78	30.67	13.11%	19.85	32.71	8.38%	16.17	25.26	10.87%	0
**STAEformer**	2.95	6.03	8.11%	1.56	3.56	3.50%	15.37	27.55	15.80%	**18.19**	30.63	**12.24%**	**19.34**	32.60	**8.10%**	**13.65**	23.57	**8.94%**	6
**Ours**	**2.94**	**6.01**	**8.10%**	**1.53**	**3.50**	**3.42%**	15.02	26.17	**15.35%**	18.37	**30.51**	12.46%	19.73	**32.11**	8.24%	**13.65**	23.84	10.34%	**10**

**Table 3:** Performance under different prediction horizons on METR-LA and PEMS-BAY.

Datasets		Metric	Ours	STAEformer	MegaCRN	GWNet	DCRNN
**METR-LA**	**Horizon 3 (15 min)**	**MAE**	**2.65**	**2.66**	**2.59**	**2.69**	**2.67**
		RMSE	5.05	5.16	**4.99**	5.15	5.21
	Horizon 6 (30 min)	MAE	**2.99**	**2.99**	**2.99**	3.07	3.08
		RMSE	6.08	**6.07**	**6.07**	6.21	6.33
	Horizon 12 (60 min)	MAE	3.37	**3.35**	3.46	3.52	3.58
		RMSE	7.12	**7.08**	7.24	7.32	7.54
**PEMS-BAY**	Horizon 3 (15 min)	MAE	**1.28**	1.32	1.30	1.31	1.31
		RMSE	**2.69**	2.80	2.71	2.77	2.76
	Horizon 6 (30 min)	MAE	**1.59**	1.63	1.61	1.67	1.65
		RMSE	**3.60**	3.67	3.63	3.84	3.78
	Horizon 12 (60 min)	MAE	**1.87**	1.90	1.89	2.02	1.98
		RMSE	**4.30**	4.31	4.37	4.73	4.65
**1^st^ Count**			**7**	4	4	0	0

Further, Table 3 presents a comparison under short-term (15-minute) and long-term (60-minute) forecasting scenarios. In 60-minute forecasts, DSSA-TCN achieved RMSE improvements of 1.4% on METR-LA and 4.3% on PEMS-BAY compared to the best-performing baselines. For the 15-minute horizon, the improvements were relatively smaller, such as a 0.9% RMSE reduction on METR-LA, while still demonstrating strong responsiveness to short-term variations. These results indicate that DSSA-TCN exhibits stronger resistance to error accumulation over longer forecasting horizons and outperforms models such as DCRNN and GWNet, whose performance degrades more significantly with increasing prediction steps. It is worth noting that the performance gap between DSSA-TCN and several baselines becomes less pronounced in short-term forecasting (e.g., 15 minutes). This observation is expected because short-term traffic dynamics are dominated by recent local trends and periodic patterns that most temporal models can effectively capture. However, DSSA-TCN demonstrates greater advantages in long-term forecasting horizons (≥60 minutes) and in datasets with higher spatial complexity, where adaptive sparse attention and bidirectional diffusion jointly enhance the modeling of long-range and directional dependencies. This finding confirms that DSSA-TCN is particularly effective for complex, multi-hop, and temporally extended traffic prediction scenarios. Among all compared methods, RNN-based models such as DCRNN exhibited relatively poor performance in long-horizon settings. This is likely due to the limited capacity of GRU encoders to capture long-range dependencies, as well as their susceptibility to gradient vanishing problems. In contrast, the convolutional architecture of DSSA-TCN provides a larger temporal receptive field and more stable optimization, resulting in better robustness and generalization across dynamic and structurally complex traffic scenarios.

### Ablation study

To evaluate the contribution of each proposed module, we conduct ablation studies by systematically removing or replacing specific components from the full model. The detailed configurations are as follows:

- **w/o Temporal Embedding:** Removes the time-of-day and day-of-week embeddings to assess the impact of temporal context modeling.
- **w/o Adaptive Embedding:** Eliminates the learnable node embeddings, retaining only structural information to evaluate the effect of personalized node representations.
- **w/o Top-k Attention:** Replaces the sparse Top-k spatial attention mechanism with full attention to examine the benefits of sparsity in attention computation.
- **w/o Diffusion GCN:** Removes the diffusion graph convolution module, relying solely on attention to model spatial dependencies, thus testing the importance of explicitly modeling topological structure.
- **Ours:** The complete model with all proposed components, serving as the performance baseline.

To evaluate the individual contributions of each model component, we conduct a comprehensive ablation study on three benchmark datasets: METR-LA, PEMS-BAY, and PEMS03. The results, summarized in Table 4, reveal several key observations. The exclusion of the diffusion graph convolution module results in the most significant performance degradation. On METR-LA, the RMSE increases from 6.01 to 6.36, which corresponds to a relative rise of 5.8%. Similarly, on PEMS03, the RMSE increases from 26.17 to 27.51, reflecting a 5.1% decrease in accuracy. These results highlight the crucial role of explicit topological modeling in capturing spatial dependencies across traffic networks. Replacing the sparse Top-k spatial attention mechanism with fully connected attention also causes noticeable declines in accuracy. For instance, on PEMS03, the RMSE increases from 26.17 to 26.85. This indicates that the sparse attention design effectively filters irrelevant spatial interactions, helping the model focus on the most informative nodes in large-scale networks. When adaptive node embeddings are removed from the model, the performance drops slightly. On PEMS03, the RMSE rises from 26.17 to 26.24. Although the decline is relatively minor, the result still confirms that incorporating node-specific representations improves predictive precision by introducing spatial heterogeneity. Removing temporal embeddings, which encode periodic patterns such as time-of-day and day-of-week, also leads to a moderate reduction in performance. On METR-LA, the RMSE increases from 6.01 to 6.08. This suggests that temporal priors contribute auxiliary information that helps the model capture recurring fluctuations more effectively. In summary, each component of DSSA-TCN contributes meaningfully to the overall performance. Among them, diffusion graph convolution and sparse attention have the most significant impact and serve as the core of spatial modeling, while temporal and adaptive embeddings offer complementary global and node-level enhancements, jointly improving the model’s generalization and stability.

**Table 4:** Ablation study of DSSA-TCN on three benchmark datasets.

Datasets	METR-LA		PEMS-BAY		PEMS03	
Metric	MAE	RMSE	MAE	RMSE	MAE	RMSE
w/o Temporal Embedding	2.98	6.08	1.56	3.58	15.10	26.73
w/o Adaptive Embedding	2.98	6.09	1.57	3.57	15.05	26.24
w/o Top-k Attention	2.96	6.07	1.55	3.53	15.25	26.85
w/o Diffusion GCN	3.06	6.36	1.55	3.56	15.57	27.51
DSSA-TCN	**2.94**	**6.01**	**1.53**	**3.50**	**15.02**	**26.17**

### Sensitivity to sparsity level

To further evaluate the impact of the sparse spatial attention mechanism on forecasting performance, we conduct a sensitivity analysis on the sparsity-level hyperparameter. Specifically, we focus on the neighbor selection ratio range used in the Top-k sparsity strategy. This mechanism employs a learnable parameter *α* to dynamically determine the number of spatial neighbors retained for each node during training. Such a design enables the model to adaptively balance spatial expressiveness and computational efficiency across different scenarios. Given the varying spatial density and topological complexity of real-world traffic graphs, the appropriate sparsity level is crucial for maintaining model stability and generalization. To this end, we perform experiments on two representative traffic volume datasets, PEMS03 and PEMS04, under six different sparsity configurations: [1/9,1/6],[1/6,1/3],[1/3,1/2],[1/2,2/3],[2/3,5/6], and a fully connected setting that retains all spatial neighbors. This analysis aims to reveal how the sparsity control mechanism influences the accuracy and robustness of the DSSA-TCN framework under different traffic conditions.

**Fig 2:**
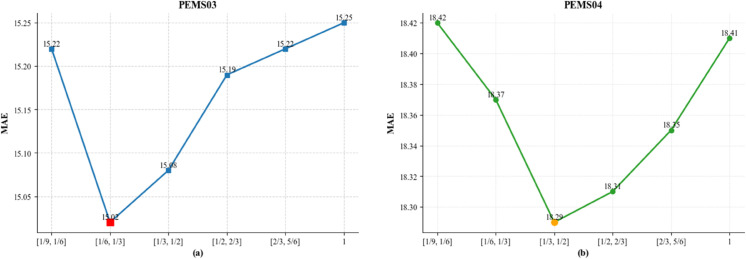
MAE performance of DSSA-TCN under varying spatial sparsity levels on the PEMS03 and PEMS04 datasets.

The results illustrated in Fig 2 reveal a consistent U-shaped relationship between model performance and spatial sparsity level. On both PEMS03 and PEMS04 datasets, the DSSA-TCN achieves the lowest MAE under moderate sparsity configurations. The optimal sparsity ranges are [1/6,1/3] for PEMS03 and [1/3,1/2] for PEMS04, suggesting that a moderate number of spatial neighbors yields the best performance. At extremely high sparsity levels, where only a minimal number of spatial neighbors are preserved, the model is deprived of adequate contextual information, hindering its ability to learn meaningful spatial correlations. This deficiency often results in underfitting, as the network fails to capture essential structural dependencies. On the other hand, reducing sparsity toward a fully connected setting introduces an abundance of irrelevant or noisy spatial signals. Such excessive connectivity not only increases computational burden but also disrupts attention stability, which can deteriorate prediction performance. These observations provide empirical support for the incorporation of a learnable sparsity mechanism. By dynamically adjusting spatial connectivity during training, the model selectively attends to the most informative nodes while suppressing redundant interactions. Proper calibration of this sparsity level is therefore crucial to fully harness the representational power of DSSA-TCN in traffic networks with diverse topological characteristics.

### Interpretability analysis of sparse attention

**Fig 3:**
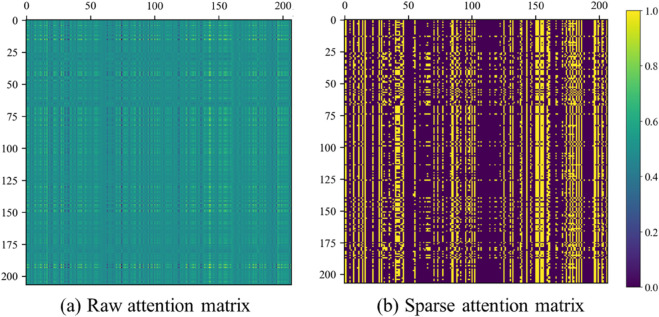
Visualization of attention matrices on the METR-LA dataset. (a) Raw attention matrix before sparsification, showing dense and noisy correlations across nodes. (b) Sparse attention matrix after the adaptive Top-*k* operation, where high-weight connections capture both local and long-range dependencies beyond the physical topology.

To assess the interpretability of DSSA-TCN, we visualize the attention maps learned in the spatial attention module. Fig 3 compares the raw attention matrix before sparsification with the sparse attention matrix after the adaptive top-*k* operation. The matrices are obtained from the final TCN block, and the attention values are averaged across heads to represent inter-node dependencies. The raw attention matrix appears dense, with many low but nonzero correlations across node pairs, which makes it difficult to identify meaningful spatial relationships. In contrast, the sparse attention matrix exhibits clear and structured patterns. Only a small subset of nodes retains high attention weights, forming visible vertical and horizontal concentration bands. These high-weight nodes correspond to major traffic sensors that exert strong global influence across the network. The visualization indicates that the adaptive sparse attention mechanism can capture both local and long-range dependencies between distant road segments that are not directly connected in the physical topology. This demonstrates that the attention mechanism complements the graph diffusion process by learning additional functional relationships beyond the predefined adjacency structure. Therefore, the sparse attention enhances model interpretability by revealing how remote regions interact dynamically within the traffic network.

### Efficiency study

**Fig 4:**
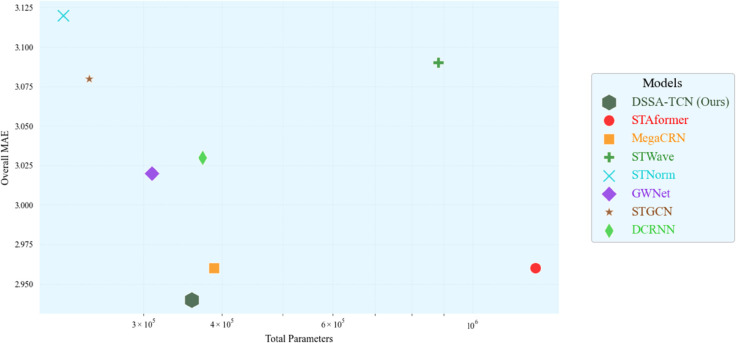
Comparison of model efficiency on the METR-LA dataset.

To evaluate the practical efficiency of the proposed model, we conduct a comparative analysis on the METR-LA dataset to examine the trade-off between predictive accuracy and model complexity. Fig 4 illustrates the relationship between the total number of trainable parameters (x-axis) and the corresponding MAE (y-axis) across all competing models. Our proposed DSSA-TCN achieves the lowest overall MAE while maintaining a relatively compact architecture with only 358,060 parameters. This highlights the model’s excellent balance between predictive performance and memory efficiency. In contrast, Transformer-based models such as STAEformer tend to incur substantially higher parameter counts due to dense attention operations over time and space dimensions. While they offer competitive accuracy, their elevated memory and computation demands may hinder practical deployment in large-scale traffic networks. DSSA-TCN, on the other hand, leverages lightweight temporal convolutions and sparse spatial attention to achieve competitive convergence with shorter per-epoch runtimes and reduced memory footprint. To further quantify the computational advantages, Table 5 reports the training time, inference latency, and parameter counts of all compared models. DSSA-TCN achieves a 19.6% faster inference speed compared to MegaCRN and maintains comparable training efficiency to lightweight baselines such as STGCN and STNorm. These results demonstrate that DSSA-TCN not only delivers state-of-the-art prediction accuracy but also exhibits excellent computational efficiency, making it a practical and scalable solution for deployment in real-world intelligent transportation systems.

**Table 5:** Computational efficiency of DSSA-TCN and baseline models on the METR-LA dataset.

Model	#Params	Training Time (s/epoch)	Inference Time (ms/step)	Relativae Speed-up vs MegaCRN(%)
DCRNN	372,353	81.65	82.23	+8.23%
STGCN	246,028	43.60	63.99	+28.6%
GWNet	309,400	78.10	28.96	+67.7%
STNorm	223,756	19.90	17.10	+80.9%
STWave	881,598	81.38	89.03	+0.63%
MegaCRN	388,761	70.08	89.59	——
STAEformer	1258,980	88.79	84.14	+10.6%
DSSA-TCN	358,060	80.53	72.01	+19.6%

## Conclusions

This paper presents DSSA-TCN, a novel spatio-temporal forecasting architecture that combines causal dilated temporal convolutions, sparse spatial attention, and diffusion graph convolution to efficiently capture complex traffic dynamics. The model is specifically designed to learn spatio-temporal dependencies at multiple scales while maintaining scalability and computational efficiency. By incorporating time-of-day embeddings, learnable node embeddings, and a Top-k sparsity strategy in the attention mechanism, DSSA-TCN achieves a favorable balance between representational expressiveness and resource efficiency. Extensive experiments on six benchmark traffic datasets, including both speed datasets such as METR-LA and PEMS-BAY and flow datasets such as PEMS03, PEMS04, PEMS07, and PEMS08, consistently demonstrate the superior or highly competitive performance of DSSA-TCN across various forecasting horizons. Ablation studies confirm the critical contributions of each architectural component, particularly the diffusion graph convolution and sparse attention mechanism. Sensitivity analysis further verifies the robustness of the model under different spatial sparsity configurations, while the efficiency study highlights DSSA-TCN’s ability to achieve state-of-the-art accuracy with substantially fewer parameters than Transformer-based counterparts.

Although DSSA-TCN achieves strong performance across diverse traffic datasets, its design fundamentally relies on explicit physical topologies such as road networks to define spatial dependencies. This assumption limits the model’s direct applicability to other spatio-temporal forecasting domains, including energy demand and weather prediction, where explicit graphs are unavailable. In such cases, spatial relations must be inferred from latent correlations or data-driven similarity matrices, which remain to be systematically explored. Addressing this limitation provides a natural direction for future research. Extending DSSA-TCN to non-physical domains through adaptive latent graph construction and examining how sparse attention and diffusion mechanisms generalize beyond transportation systems will further enhance its universality and robustness.

Future work may focus on enriching the model input with multimodal contextual information such as road occupancy, incident reports, and weather conditions. In addition, extending the framework to accommodate dynamically evolving traffic graphs and incorporating continual learning strategies would enhance its adaptability in real-time deployments. Finally, we aim to generalize the model towards a unified architecture capable of jointly predicting multiple traffic variables such as speed, flow, and occupancy for comprehensive urban mobility forecasting.
